# Severe Diverticulitis With Streptococcus anginosus Bacteremia Complicated by Native Hip Septic Arthritis and Subacromial Bursitis

**DOI:** 10.7759/cureus.104129

**Published:** 2026-02-23

**Authors:** Salah Daghlas, Nasam AlTwal, Fernando Merino

**Affiliations:** 1 Internal Medicine, University of Kansas Health System, Kansas City, USA; 2 Psychiatry, University of Kansas Health System, Kansas City, USA; 3 Infectious Diseases, University of Kansas Health System, Kansas City, USA

**Keywords:** diverticulitis, gram positive bacteremia, hepatic abscess, septic arthritis, streptococcus anginosus, subacromial bursitis

## Abstract

A 38-year-old man was admitted for severe diverticulitis complicated by *Streptococcus anginosus* bacteremia. A CT of the abdomen and pelvis on admission was significant for the additional finding of several hepatic abscesses and a small left hip effusion. On examination, the passive and active range of motion of his left hip and shoulder was limited and painful. A hepatic abscess drain was placed, with aspirate cultures yielding *Streptococcus anginosus*. Additionally, a left hip arthrocentesis was performed with cultures yielding *Streptococcus anginosus*. A left shoulder arthrocentesis did not yield any fluid; however, an MRI was relevant for synovial thickening with septation of an enlarged subacromial bursa, suggestive of subacromial bursitis. The patient underwent an arthrotomy with lavage of the left hip joint and completed a four-week course of antibiotic therapy with resolution of the infection and hip and shoulder pain. This is a case describing *Streptococcus anginosus*, an organism typically associated with pyogenic infections, causing native hip septic arthritis and probable septic subacromial bursitis. This highlights *Streptococcus anginosus*' potential to cause musculoskeletal infections.

## Introduction

*Streptococcus anginosus* is a gram-positive coccus that is part of the *Streptococcus anginosus* group, also referred to as the *Streptococcus milleri* group. Other members of this group are *Streptococcus intermedius* and *Streptococcus constellatus*. *Streptococcus anginosus* is typically found in oropharyngeal and gastrointestinal flora [1,2]. It has previously been considered a commensal organism; however, it has been increasingly viewed as an opportunistic organism with a propensity for intrabdominal pyogenic infections [2,3]. The mechanisms underlying this propensity are poorly understood. One contributing mechanism is an anchoring protein, referred to as fibronectin-binding protein, that enables *Streptococcus anginosus* to adhere to host tissue and cause pyogenic infections [4]. An additional possible mechanism includes the presence of special nucleases that allow it to evade neutrophil extracellular traps, which are composed of nucleic acids [5]. We present the case of a patient who was found to have diverticulitis with *Streptococcus anginosus* bacteremia complicated by proven left hip septic arthritis and probable left septic subacromial bursitis with suggestive imaging findings. 

## Case presentation

A 38-year-old man initially presented to an urgent care clinic with a chief complaint of left hip and shoulder pain. Past medical history included hypertension, non-insulin-dependent type 2 diabetes, and diverticulosis. He denied any traumatic falls or previous injuries to his hip or shoulder. Further history revealed that he had several days of loose bowel movements, malaise, and poor oral intake. He reported that his symptoms, including his joint pain, developed five days prior to presentation. At that time, he denied significant abdominal pain. He was sent to the emergency department for further evaluation.

**Figure 1:**
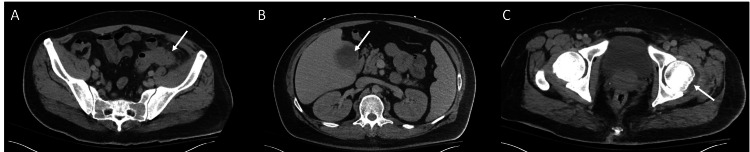
Axial CT of the abdomen and pelvis with contrast showed severe sigmoid diverticulitis with hepatic fluid collection and small left hip joint effusion. (A) Sigmoid diverticulitis with associated fat stranding; (B) Hepatic abscess; (C) Small left hip joint effusion; Arrows point to respective processes (A-C).

His initial vital signs included a blood pressure of 94/54 mmHg, a heart rate of 93 beats per minute, an oxygen saturation of 100%, a respiratory rate of 20 breaths per minute, and a temperature of 36.7°C. His laboratory studies were significant for a white blood cell count of 21 10^3^/uL (normal range 4.5-11 10^3^/uL). Other laboratory findings are found in Table 1. A CT of the abdomen and pelvis with contrast was obtained, which showed severe complicated sigmoid diverticulitis with a 2 cm left abdominal wall abscess. Notable findings included multiple hepatic abscesses and a small left hip effusion (Figure 1). Blood cultures were obtained. He was admitted to the inpatient surgical service for further management of his diverticulitis. Piperacillin/tazobactam 4.5 grams every six hours was started. 

**Table 1:** The patient's initial serum laboratory values on admission

Serum Laboratory Test	Values (reference range)
White blood cell count	21 (4.5-11 10^3^/uL)
Sodium	127 (137-147 mmol/L)
Creatinine	2.84 (0.4-1.24 mg/dL)
Aspartate aminotransferase	59 (7-40 U/L)
Alanine aminotransferase	41 (7-56 U/L)
Alkaline phosphatase	208 (25-110 U/L)
Bilirubin	1.4 (0.3-1.2 mg/dL)
Point-of-care lactate	0.89 (0.5-2 mmol/L)

The Infectious Diseases team was consulted on the second day of admission due to a concern for left hip septic arthritis in the setting of the noted hip joint effusion and severe sepsis from diverticulitis. Of note, the patient reported an episode of diverticulitis that was managed in another hospital months before this admission. He had a colonoscopy shortly after that admission, which did not show evidence of malignancy. 

The patient again denied significant abdominal pain; however, he noted that he was most concerned about his left hip and shoulder pain. He denied any hardware in either the left hip or shoulder. Examination was limited by severe pain with passive and active range of motion for both the left hip and shoulder. It was recommended that he undergo arthrocentesis of the left hip and further imaging of the left shoulder. Shortly after his admission, his blood cultures grew *Streptococcus anginosus* in four out of four bottles with susceptibility to ceftriaxone and penicillin. Antibiotics were de-escalated to ceftriaxone 2 grams daily and metronidazole 500 mg twice a day. He did reach a maximum temperature of 39.3°C on his second day of admission; however, he became afebrile for the rest of the admission shortly after. Repeat blood cultures, obtained 48 hours after initial blood cultures, did not grow any organisms. A dental panoramic X-ray was obtained, which did not show dental caries. A transthoracic echocardiogram did not show any vegetations. 

A hepatic abscess drain was inserted in the largest hepatic abscess. Aspirate cultures grew *Streptococcus anginosus* without the presence of other organisms. 

**Figure 2:**
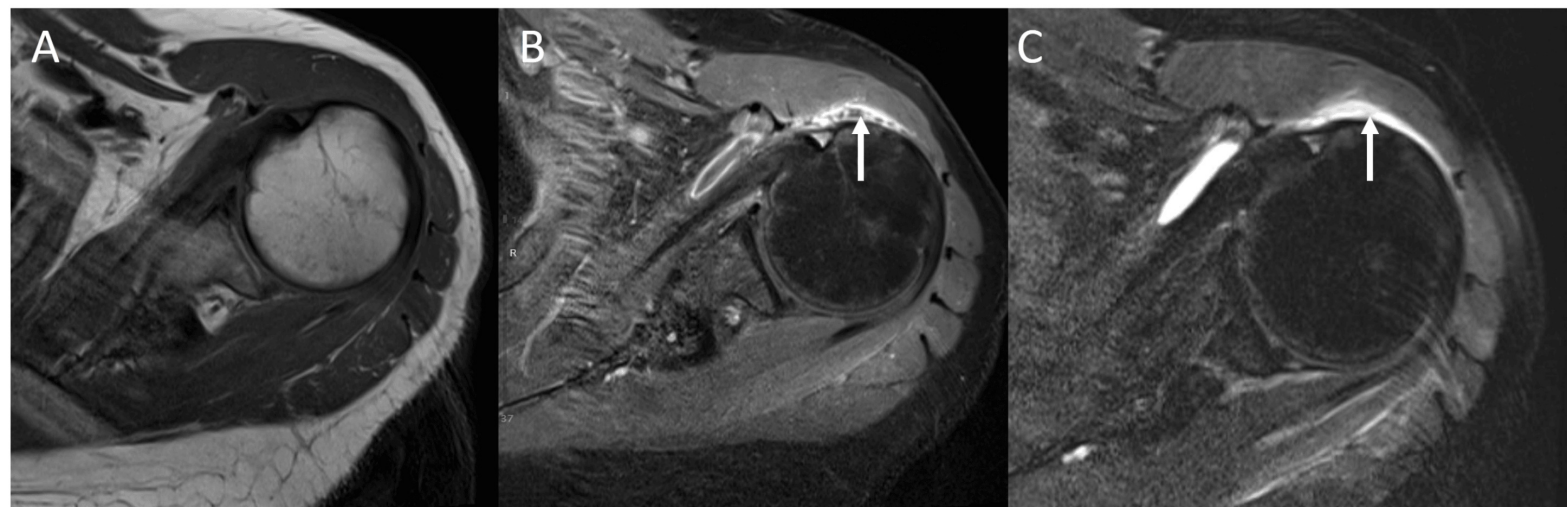
Left shoulder MRI with and without contrast demonstrated subacromial bursitis (A) Axial T1 phase pre-contrast; (B) Axial T1 phase post-contrast demonstrated septation; (C) Axial T2 phase demonstrated synovial thickening and oedema within the subacromial bursa; arrows point to respective features (B-C).

Arthrocentesis of the left hip was performed, and synovial fluid studies are described in Table 2. Orthopaedics was consulted for a left hip joint lavage and arthrocentesis of the left shoulder due to rising concern for seeding of *Streptococcus anginosus* in both joints. During the left hip arthrotomy, approximately 20 mL of purulent synovial fluid was encountered. Fluid was unable to be obtained in the left shoulder joint; thus, it was recommended to obtain an MRI of the left shoulder. It was significant for subacromial bursitis (Figure 2); however, bursal fluid was not aspirated as it was decided it would not change medical management. Several days later, the left hip synovial fluid cultures grew *Streptococcus anginosus*. 

**Table 2:** Left hip synovial fluid studies

Synovial Cell Type	Cell Count (cells/uL)/Percentage (%)
Total white blood cells	55,245 (reference range: 0-200)
Polymorphonuclear leukocytes	52,483 (95%; reference %: <25%)
Red blood cells	23,295 (reference range: 0)

Repeat CT of the abdomen and pelvis imaging showed a persistent size of the draining hepatic abscess, and a decision was made to upsize his hepatic drain. By the end of his admission, the patient’s hip and shoulder pain had improved, and his white blood cell count, liver, and kidney function normalised. A peripherally inserted central catheter was placed, and he was discharged on a total of two weeks of ceftriaxone 2 grams daily, from the time of source control, followed by two weeks of oral linezolid 600 mg twice a day. The choice to do two weeks of parenteral therapy followed by two weeks of oral therapy was based on expert opinion [6,7]. The patient was scheduled and instructed to follow up with the Infectious Diseases clinic. 

Three weeks after discharge, he underwent an abscessogram, which showed near-complete resolution of the hepatic abscess being drained. Drain removal in the general Surgery clinic was performed the following week. Nearly two months after discharge, in a follow-up appointment with orthopaedics, the patient noted that mobility of his left hip and shoulder had essentially returned to baseline.

## Discussion

We describe the case of a 38-year-old man with diverticulitis with *Streptococcus anginosus* bacteremia complicated by seeding into the left hip joint and likely the left subacromial bursa. Typically, *Streptococcus anginosus*-associated infections have a predilection for causing pyogenic infections. This is consistent with liver abscess formation in our patient. This is most likely caused by haematogenous dissemination in the setting of bacteremia [2]. However, *Streptococcus anginosus'* association with joint infections is not as common or as well described. 

Briefly, septic arthritis should be suspected in patients presenting with acute monoarticular joint pain, especially in the presence of fever, bacteremia, or erythema and swelling with limited range of motion. Risk factors include older age, pre-existing joint disease, immunosuppression, diabetes mellitus, and prosthetic joints. The probability of septic arthritis increases with higher thresholds for synovial white blood cell count. Notably, a synovial WBC count of 50,000 cells/uL yields a positive likelihood ratio of 7.7. Additionally, a neutrophil composition greater than 90% yields a positive likelihood ratio of 3.4 [8]. Of note, our patient is diabetic and had a synovial WBC greater than 50,000 cells/uL composed of greater than 90% neutrophils. 

There have been reported cases of septic arthritis secondary to *Streptococcus anginosus*; however, this has largely been limited to case reports [9-12]. Risk factors in these reports ranged from pre-existing structural damage, including osteoarthritis and rheumatoid arthritis, to immunocompromising factors such as alcoholism and diabetes. Additionally, three retrospective studies highlight the rare incidence of *Streptococcus anginosus *group organisms causing septic arthritis, with 1% to 4% of cases attributed to this group. Joint locations affected by the *Streptococcus anginosus* group are not specified in two studies [13,14], while the other study evaluated the incidence of spinal facet joint septic arthritis [15]. 

Considering our patient had atraumatic left hip and left shoulder pain occurring around the same time, both with limitation of range of motion, it is probable that the left subacromial bursa was also infected. Imaging supports this assumption, as there was radiographic evidence of bursitis on MRI imaging. This conclusion is further supported by the resolution of left shoulder pain with the completion of antibiotics. It is somewhat atypical that bursitis presented with significant limitations to passive range of motion, in addition to expected limitations to active range of motion. However, this type of presentation in subacromial bursitis has been previously reported [16]. To our knowledge, this is the second reported case of subacromial bursitis thought to be secondary to *Streptococcus anginosus* [17]. The patient in that case had a history of diabetes and osteoarthritis. 

## Conclusions

This report features a case of *Streptococcus anginosus* causing a pyogenic intra-abdominal infection, a well-recognized complication caused by this organism, with bacteremia. Importantly, the case illustrates the value of a comprehensive physical exam in patients with bacteremia. This prompted further evaluation of the left hip and shoulder, leading to a diagnosis of left hip septic arthritis and probable left subacromial septic bursitis. Ultimately, this report adds to the existing literature regarding *Streptococcus anginosus’* ability to cause musculoskeletal infections.
